# Plant Biostimulants: New Insights Into the Biological Control of Verticillium Wilt of Olive

**DOI:** 10.3389/fpls.2021.662178

**Published:** 2021-05-20

**Authors:** Ana López-Moral, Carlos Agustí-Brisach, Antonio Trapero

**Affiliations:** Departamento de Agronomía (Excellence Unit ‘María de Maeztu’ 2020-23), ETSIAM, Universidad de Córdoba, Córdoba, Spain

**Keywords:** biocontrol, biostimulants, *Olea europaea*, resistance inductors, *Verticillium dahliae*

## Abstract

Verticillium wilt of olive (*Olea europaea* subsp. *europaea* L.) (VWO), caused by the hemibiotrophic soil-borne fungus *Verticillium dahliae* Kleb., is considered the major limiting factor of this crop in Mediterranean-type climate regions of the world. The absence of effective chemical treatments makes the control of the disease difficult. In this way, the use of biostimulants and host plant defense inducers seems to be one of the most promising biological and eco-friendly alternatives to traditional control measures. Thus, the main goal of this study was to evaluate the effect of 32 products, including amino acids, micronutrients, microorganisms, substances of natural origin, copper complex-based products, and organic and inorganic salts against the disease under controlled conditions. To this end, their effects on mycelial growth and microsclerotia (MS) inhibition of *V. dahliae* were evaluated by means of dual cultures or by sensitivity tests *in vitro* as well as on disease progression *in planta*. Wide ranging responses to the pathogen and disease reduction levels were observed among all the products tested, suggesting multiple modes of action. Copper-based products were among the most effective for mycelial growth and MS inhibition, whereas they did not show an important effect on the reduction of disease severity *in planta*. *Phoma* sp. and *Aureobasidium pullulans* were the most effective in disease reduction *in planta* with foliar application. On the other hand, two phosphite salts, one with copper and the other with potassium, were the most effective in disease reduction *in planta* when they were applied by irrigation, followed by *A. pullulans* and *Bacillus amyloliquefaciens*. This study will be useful to select the best candidates for future studies, contributing significantly to new insights into the current challenge of the biological control of VWO.

## Introduction

Verticillium wilt of olive (*Olea europaea* subsp. *europaea* L.) (VWO) is considered the major limiting factor of this crop in Mediterranean-type climate regions of the world since it causes high levels of tree mortality and fruit yield reduction (Montes-Osuna and Mercado-Blanco, 2020). The disease is caused by the hemibiotrophic soil-borne fungus *Verticillium dahliae* Kleb. Two populations of the pathogen, defoliating (D) and nondefoliating (ND) pathotypes, have been well distinguished in olive, with the D pathotype causing the most severe damage. In any case, the pathogen is characterized by the production of infective propagules known as microsclerotia (MS), which are dormant structures that allow the fungus to survive in the soil for long periods of time (López-Escudero and Mercado-Blanco, 2011; Jiménez-Díaz et al., 2012; Montes-Osuna and Mercado-Blanco, 2020).

The best strategies for the management of VWO should be focused on reducing the survival of MS in the soil as well as preventing their germination (Antonopoulos et al., 2008). However, the ability of MS to survive for up to 14 years in the soil and to infect a broad diversity of alternative hosts, added to the absence of effective chemical treatments against the disease, makes VWO control difficult (Trapero et al., 2015). Therefore, an integrated disease management (IDM) strategy including both pre- and postplanting treatments must be strongly considered for the control of the disease within the framework of sustainable agriculture (López-Escudero and Mercado-Blanco, 2011; Montes-Osuna and Mercado-Blanco, 2020; Ostos et al., 2020). In this framework, the implementation of natural products such as essential oils or organic amendments (OAs) as well as biological control agents (BCAs) against VWO has been studied by several authors over the last two decades as a potential eco-friendly control measure against the disease (Montes-Osuna and Mercado-Blanco, 2020). In fact, the search for novel BCAs, including bacteria [*Pseudomonas* spp. (Ruano-Rosa et al., 2017; Gómez-Lama Cabanás et al., 2018b), strains of the Bacillales order (Tjamos et al., 2004, 2005; Markakis et al., 2016; Gómez-Lama Cabanás et al., 2018a; Azabou et al., 2020), etc.] and fungi [i.e., *Aureobasidium* spp.; *Phoma* sp. (Varo et al., 2016); nonpathogenic *Fusarium oxysporum* strains (Mulero-Aparicio et al., 2019a,b, 2020); *Trichoderma* spp. (Carrero-Carrón et al., 2016, 2018; Ruano-Rosa et al., 2016; Morán-Diez et al., 2019), etc.] have led to promising results against VWO. However, most of these studies have been conducted under controlled experimental conditions, with the exception of those conducted by Markakis et al. (2016) and Mulero-Aparicio et al. (2020). These authors evaluated the efficiency of the BCA *Paenibacillus alvei* strain K165 (Markakis et al., 2016) and the nonpathogenic *F. oxysporum* strain FO12 (Mulero-Aparicio et al., 2020) in suppressing VWO in naturally infected fields with promising results. However, further research is needed to develop future biological preformulations for their commercialization.

In addition to the BCAs described above, more than 230 natural products, including OAs, microorganisms, plant extracts, essential oils, and mixtures of them, have also been evaluated in recent years to determine their effectiveness in suppressing *V. dahliae* under controlled conditions (Lozano-Tovar et al., 2013; Varo et al., 2016, 2017, 2018). The most effective products derived from these last studies have also been evaluated under experimental field conditions in naturally infested soils by Mulero-Aparicio et al. (2020), who showed that a commercial essential oil from *Thymus* sp. and the grape marc compost CGR03 were able to significantly reduce the disease incidence.

Recently, the use of seaweeds such as alginate, laminarin, carrageenan and ulvan in the biological control of VWO has also been evaluated. Seaweeds are considered potential elicitors of phenylalanine ammonia-lyase (PAL) and lignin contents, which could markedly reduce vascular discoloration in affected olive twigs (Salah et al., 2018). Likewise, it is worth mentioning that the use of biostimulants and host plant defense inducers (HPDI) also seems among the most promising biological and ecofriendly alternatives to traditional control measures (Sharma et al., 2014; Drobek et al., 2019; Barros-Rodríguez et al., 2020). It has been demonstrated that plants are able to activate a battery of defensive responses against biotic or abiotic stresses when they are previously stimulated by means of appropriate natural or chemical products (Conrath, 2009; Llorens et al., 2017b). From a phytopathological point of view, these stimuli are interesting since they not only induce plant innate resistance that could be enough to overcome the attack of the pathogen but also persist in the plant for several months with long-term effects preventing new infections (Bektas and Eulgem, 2015; Llorens et al., 2017b). In addition, these products have low or null toxicity, contributing to a reduction in the number of residues in fruit and vegetables (González-Hernández et al., 2018). For all these reasons, biostimulants and HPDI are advantageous in term of sustainability, and their use is allowed in IDM strategies (González-Hernández et al., 2018; Montes-Osuna and Mercado-Blanco, 2020).

Because this kind of products could be a novel and potentially eco-friendly alternative control measure for VWO in IDM strategies, the main goal of this study was to evaluate the effect of 32 products grouped under the terms BCAs, biostimulants, HPDI, and fungicides against the disease under controlled conditions. These last were included for comparative purposes. Most of the products included in this study (22 out of 32; Table 1) were carefully selected for their potential activity as plant biostimulants according to the most recent European regulatory framework on this topic [European Regulation (EU) 2019/1009], which defines plant biostimulants as “*a product stimulating plant nutrition processes independently of the product’s nutrient content with the sole aim of improving one or more of the following characteristics of the plant or the plant rhizosphere as (a) nutrient use efficiency, (b) tolerance to abiotic stress, (c) quality traits and d) availability of confined nutrients in soil or rhizosphere.*” First, the effect of the 32 selected products on mycelial growth and MS inhibition of *V. dahliae* was evaluated by means of dual cultures or by sensitivity tests *in vitro*. This first step was useful to check whether the products had any fungicidal effect and interfered with the growth or survival of the pathogen. Subsequently, the potential biocontrol effects of all the products were also evaluated on the progress of the disease in olive plants inoculated with the pathogen. This study will be useful to select better candidates for future studies that should be conducted under the EU 2019/1009, contributing significantly to new insights into the current challenge of the biological control of VWO.

**TABLE 1:** Biostimulants, resistance inductors and biological products evaluated against VWO.

Active ingredient(s)	Trade name/Formulation^a^	Manufacturer	Class (FRAC code)^b^	Mode of action^c^	Dose^d^	
					Foliar	Irrigation
***Non-commercial products***						
*Aureobasidium pulullans*	AP08	DAUCO^e^	Fungal (NC)	BCA	10^6^ conidia/ml	10^6^ conidia/ml
*Bacillus amyloliquefaciens*	PAB-024	DAUCO^e^	Bacterial (BM02)	BCA	10^8^ CFU/ml	10^8^ CFU/ml
*Phoma* spp.	ColPat-375	DAUCO^e^	Fungal (NC)	BCA	10^6^ conidia/ml	10^6^ conidia/ml
***Commercial products***						
*Bacillus subtilis*	Serenade^®^ -WG	Bayer CropScience	Bacterial (BM02)	BCA/FG	4 g/l	4 g/l
Fungal extracts	Cybelion^®^ -EW	Adama	Natural compound (NC)	PB	3 ml/l	3 ml/l
Seaweed extracts (*Laminaria digitata*)	Vacciplant Max^®^ -EW	UPL	Natural compound (P04)	PB	1 ml/l	1 ml/l
Bioassimilable sulfur 1	Naturdai S-System^®^ -EW	Idai Nature	Inorganic (M02)	PB	5 ml/l	3 ml/l
Bioassimilable sulfur 2	Thiopron^®^ -EW	UPL	Inorganic (M02)	PB	7 ml/l	7 ml/l
Copper chloride	Copper (II) chloride-SL	Panreac	Inorganic (M01)	FG	5.36 g/l	5.36 g/l
Copper complexed 1	Bioscrop Acticuper^®^ -EW	Econatur	Inorganic (M01)	PB	2 ml/l	2 ml/l
Copper complexed 2	Disper Cu Max^®^ -WG	Disper	Inorganic (M01)	PB	1.5 g/l	2 g/l
Copper gluconate 1	Glucopper^®^ -EW	Tradecorp	Inorganic (M01)	PB	3 ml/l	12 ml/l
Copper gluconate 2	Idai Cobre^®^ -EW	Idai Nature	Inorganic (M01)	PB	4 ml/l	3 ml/l
Copper sulfate	Copper sulfate-SL	Merck	Inorganic (M01)	FG	8 g/l	8 g/l
Hydrogen peroxide	Huwa-San 50 Agro^®^ -SL	Huwa-San España	Inorganic compound (NC)	FG	0.1 ml/l	0.1 ml/l
Aluminum lignosulfonate	Brotaverd^®^ -EW	Idai Nature	Inorganic salt (NC)	PB	5 ml/l	5 ml/l
Potassium silicate 1	Green Silk^®^ -EW	Agrinova	Inorganic salt (NC)	PB	2.5 ml/l	8 ml/l
Potassium silicate 2	Silicasol^®^ -EW	BC Fertilis	Inorganic salt (NC)	PB	3 ml/l	8 ml/l
Copper phosphite 1	Copper phosphite^®^ -EW	Nufol	Phosphorous acid and salts (P07)	PB	3 ml/l	10 ml/l
Copper phosphite 2	Naturfos Cu^®^ -EW	Daymsa	Phosphorous acid and salts (P07)	PB	3 ml/l	10 ml/l
Copper phosphite 3	Phoscuprico^®^ -EW	Agrinova	Phosphorous acid and salts (P07)	PB	3 ml/l	10 ml/l
Fosetyl-Al	Aliette^®^ -WG	Bayer CropScience	Phosphorous acid and salts (P07)	HPDI	3 g/l	10 g/l
Potassium phosphite 1	Fitasio^®^ -EW	Agrinova	Phosphorous acid and salts (P07)	PB	3 ml/l	7 ml/l
Potassium phosphite 2	Long life^®^ -EW	Nufol	Phosphorous acid and salts (P07)	PB	2 ml/l	7 ml/l
Potassium phosphite 3	Naturfos^®^ -EW	Daymsa	Phosphorous acid and salts (P07)	PB	3 ml/l	8 ml/l
Potassium phosphite 4	Alexin 75^®^ -EW	Massó	Phosphorous acid and salts (P07)	PB	4 ml/l	4 ml/l
Amino acids	AminoPhos^®^ -EW	Daymsa	Organic compound (NC)	PB	3 ml/l	8 ml/l
Amino acids + Cu	Nanocrop Cobre^®^ -EW	Agrostock	Organic compound (NC)	PB	3 ml/l	3 ml/l
Amino acids + N, P, K, and S	Daluben^®^ -WG	Folgrant S.L.	Organic compound (NC)	PB	5 g/l	5 g/l
Chitosan	Biofender Fusarum^®^ -EW	Econatur	Organic compound (NC)	HPDI	2.5 ml/l	2.5 ml/l
Organic carbon	Organihum Plus^®^ -EW	Econatur	Organic compound (NC)	PB	0.5 ml/l	1.5 ml/l
Salicylic acid	Salicylic acid^®^ -SL	Sigma-Aldrich	Organic acid (NC)	HPDI	5 mM (0.69 g/l)	5 mM (0.69 g/l)

*^*a*^EW, emulsion: oil in water; SC, suspension concentrate; SL, soluble concentrate; WG, water dispersible granule. ^*b*^Group and code numbers are assigned by the Fungicide Resistance Action Committee (FRAC) according to different modes of actions (NC, not classified; for more information, see http://www.frac.info/). ^*c*^Mode of action known currently for each product, although we cannot discard that they can act by means another mode of action. BCA (Biological Control Agent); PB (Plant Biostimulant; selected according to manufacturer instructions, based on European Regulation 2019/1009); HPDI (Host Plant Defense Inducers; selected according to FRAC); Fungicides (FG, selected according to manufacturer instructions); n/d, non determined. ^*d*^Maximum foliar or irrigation dosage recommended for the manufacturers of the commercial products was used in this study. Fungal and bacterial inocula from the BCAs (AP08, PAB-024, and ColPat-375) were prepared and adjusted according to Varo et al. (2016). ^*e*^Microorganisms (BCAs) conserved in the collection of the Department of Agronomy at the University of Córdoba (DAUCO, Spain).*

## Materials and Methods

### Fungal Isolate and Culture Conditions

*Verticillium dahliae* strain V180 isolated from soil samples collected from a commercial olive orchard affected by VWO in Villanueva de la Reina (Jaen province, southern Spain) was used in all the experiments. This isolate was previously characterized as the D pathotype by PCR. It was highly virulent in inoculated olive plants and was maintained as a single-spore isolate on potato dextrose agar (PDA; Difco Laboratories, MD, United States) slants fully filled with sterile paraffin oil at 4°C in darkness in the collection of the Department of Agronomy at the University of Córdoba (DAUCO, Spain). Prior to conducting each experiment described below, fresh colonies of the V180 strain were obtained from the collection by plating small mycelial fragments of the colonized agar from the tube onto PDA acidified with lactic acid [APDA; 2.5% (vol/vol) at 2.5 ml/litre of medium] and incubated at 24°C in darkness for 10 days. Subsequently, fresh colonies were transferred to PDA, incubated as described before and then used as inoculum sources.

### Products

A total of 32 products, including amino acids, micronutrients, microorganisms, substances of natural origin, copper complex-based products, and organic and inorganic salts, were evaluated. Twenty-nine of the 32 products were commercial products, and they were tested at the dose indicated by the respective manufacturers for either foliar or irrigation applications (Table 1). The three noncommercial products tested were microorganisms that were selected as potential BCAs from the collection of the Department of Agronomy at the University of Córdoba (DAUCO, Spain). These included two fungi [*Aureobasidium pullulans* isolate AP08 (from a leaf of *O. europaea* cv. Picual) and *Phoma* sp. isolate ColPat-375 (from xylem vessels of *O. europaea* cv. Arbequina)] and one bacterium (*Bacillus amyloliquefaciens* isolate PAB-24; from buds of *Pistacia vera* cv. Kerman). The fungal isolates were conserved as described before, and the bacterial isolate was cryopreserved with 30% glycerol at −80°C. Fungal and bacterial inocula of the BCAs were prepared and adjusted according to Varo et al. (2016).

### Effect on Mycelial Growth

#### Dual Culture Assays

The three potential BCAs described above were tested for antagonism against the *V. dahliae* isolate V-180 by means of dual culture on PDA. For this purpose, a mycelial plug (7.5 mm in diameter) of the pathogen was taken from the edge of a 7-day-old actively growing colony and plated 2 cm beyond the border of a Petri dish (9 cm in diameter) filled with PDA. In this same position, but on the other side of the Petri dish, one mycelial plug (7.5 cm in diameter) of the fungal isolates AP08 or ColPat-375 obtained from the edge of a 7-day-old actively growing colony or a single straight plug from a 2-day-old colony of the bacterial isolate PAB-24 was placed. Additionally, a mycelial plug of *V. dahliae* isolate V-180 was plated alone on PDA as a control. All Petri dishes were incubated at 24°C for 14 days in darkness. The experiment was conducted twice, and a randomized complete block design with four replicated Petri dishes per BCA was used. After 14 days of incubation, the largest and smallest diameters of the colonies of *V. dahliae* were measured, and the mean data were converted to obtain the mycelial growth rate (MGR, mm day^–1^). The mycelial growth inhibition percentage [mycelial growth inhibition, MGI (%)] was calculated as follows:

$$\mathrm{MGI}=\left[1-\left({\mathrm{MGR}}_{\mathrm{dc}}/{\mathrm{MGR}}_{\mathrm{control}}\right)\right]\times 100$$

where “MGR_*dc*_” is the MGR of *V. dahliae* in dual cultures with BCAs, and “MGR_*control*_” is the MGR of *V. dahliae* alone.

#### *In vitro* Sensitivity Tests

The effect of the twenty-nine remaining products on the mycelial growth of *V. dahliae* isolate V-180 was examined by sensitivity tests on PDA. Because the concentration of the main active ingredient was not specified by the manufacturers for most of the products evaluated in this study (mainly biostimulants and resistance inductors), we were not able to calculate specific concentrations according to the active ingredient. Thus, the following three levels of dosage were established: (i) high: the maximum dose recommended by the manufacturer of each product for its application by irrigation (Table 1); (ii) medium: 1/4 of the high dose; and (iii) low: 1/16 of the high dose. To this end, the appropriate volume (ml/l) or weight (g/l) of each product was added to sterilized PDA at approximately 45°C to achieve the required dose and poured into 9-cm diameter Petri dishes. After solidification, a mycelial plug (7.5 mm in diameter) of *V. dahliae* isolate V-180 obtained from the edge of a 7-day-old actively growing colony was placed in the center of the Petri dish. Additionally, a mycelial plug of *V. dahliae* isolate V-180 was also placed in the center of a nonamended PDA Petri dish as a control. Petri dishes were incubated for 14 days as described before. There were four replicated Petri dishes per product and dose combination. A factorial design with two independent factors (29 products and three doses per product) was used (29 × 3 × 4 = 348 Petri dishes in total). The experiment was conducted twice. The MGR (mm day^–1^) was obtained, and the MGI (%) was calculated for each product and dose combination as follows:

$$\mathrm{MGI}=\left[1-\left({\mathrm{MGR}}_{\mathrm{treatment}}/{\mathrm{MGR}}_{\mathrm{control}}\right)\right]\times 100$$

where “MGR_*treatment*_” is the MGR of *V. dahliae* on PDA amended with the respective treatment, and “MGR_*control*_” is the MGR of *V. dahliae* on non-amended PDA.

#### Effect on Microsclerotia Viability

Soil samples were collected from a commercial cotton (*Gossypium hirsutum* L.) field naturally infested with *V*. *dahliae*, located in Villanueva de la Reina (Jaen province, Andalusia region, southern Spain; Geographic coordinates 38°00’10.8”N 3°55’57.5”W). Five soil subsamples (5,000 g each) were randomly collected across the field from the upper 30 cm. Once in the laboratory, the samples were mixed to obtain a single homogenized sample, which was air-dried at room temperature until completely dry and manually sifted through a 0.8 mm-diameter sieve to remove organic debris and large particles (Trapero et al., 2013).

The experiment was conducted using sterile plastic pots (100 ml vol.) with holes previously drilled in the base (5 holes, 2 mm in diameter each) to facilitate percolation. Subsequently, each plastic pot was filled with 60 g of naturally infested air-dried soil and irrigated with 30 ml of the treatment suspension. Treatments were performed using the irrigation dose indicated for each product in Table 1. An additional plastic pot filled with 60 g of naturally infested soil was irrigated with sterile distilled water and used as a positive control. After the treatment percolated, the plastic pots were hermetically closed and incubated for 24 h at room temperature. After incubation, treated soil samples were removed from the plastic pots, deposited in individual aluminum trays, and air-dried at room temperature for 10–14 days. A completely randomized design was used with three replicated plastic pots for each product or control treatment (33 × 3 = 99 plastic pots in total). The experiment was conducted twice.

The inoculum density of *V. dahliae* expressed as the number of colony forming units (CFU) or microsclerotia per g of soil (MS) in each treated soil sample was estimated by wet sieving (Huisman and Ashworth, 1974) using 10 replicated Petri dishes of modified sodium polypectate agar medium (MSPA) (Butterfield and DeVay, 1977) following the protocol described by Varo et al. (2016). Subsequently, the percentage of inoculum density reduction was calculated with respect to the control and is expressed as MS inhibition (MSI, %).

### Effect on Verticillium Wilt Development in Olive Plants

#### Plant Material, Inoculum Preparation, and Inoculation

Healthy 6-month-old rooted olive cuttings of cv. Picual (highly susceptible to *V. dahliae*; López-Escudero et al., 2004) growing in peat moss in plastic pots (0.5 l) were obtained from a commercial nursery. To induce the active growth of the plants, they were maintained in a controlled-growth chamber [22 ± 2°C, with a 14:10-h (light:dark) photoperiod of white fluorescent light (10.000 lux) and 60% relative humidity (RH)] for 1 month before conducting the experiment, and they were irrigated three times per week.

For inoculum preparation, 2-l Erlenmeyer flasks were filled with 1 kg of a cornmeal-sand mixture (sand, cornmeal and distilled water; 9:1:2, weight:weight:volume) and double-sterilized on two consecutive days at 120°C for 50 min (1st day) and 120°C for 20 min (2nd day). Flasks were manually shaken between the two sterilizations. Subsequently, 50 mycelial plugs (7.5 mm in diameter) of *V. dahliae* isolate V180 growing on PDA as described before were introduced into each flask, and the flasks were incubated at 24°C in darkness for 4 weeks. To favor the homogeneous colonization of the cornmeal-sand mixture by the pathogen, flasks were manually shaken just after inoculation as well as once a week during the incubation period. After 4 weeks of incubation, the inoculum density of the colonized cornmeal sand was estimated by means of the serial dilution method on PDA and expressed as colony-forming units (CFUs) (Mulero-Aparicio et al., 2019a).

At the inoculation time, olive plants were transplanted to plastic pots (0.8 l) previously disinfested with a commercial sodium hypochlorite solution at 20% for 2 h and filled with a 20% (weight/weight) mixture of colonized corn meal-sand and sterile peat moss (theoretical inoculum density of the final substrate = 10^7^ CFU g^–1^; Mulero-Aparicio et al., 2019a). Additionally, olive plants transplanted into plastic pots filled with a 20% (weight/weight) mixture of sterile corn meal-sand and sterile peat moss were used as negative controls. All plants were incubated in a growth chamber at 20°C in darkness and 100% relative humidity (RH) for 7 days. Subsequently, light and humidity parameters were progressively modified over 1 week until reaching 23°C, a 12-h photoperiod of fluorescent light [10,000 lux] and 70% RH, which were maintained until the end of the experiment. Plants were irrigated three times per week.

#### Plant Treatment and Experimental Design

All the products included in this study were evaluated *in planta* by foliar spray and irrigation application. The doses used for each type of application are shown in Table 1. The foliar and irrigation applications were made by spraying 15 ml per plant or by irrigation with 350 ml per plant of the dilutions of each product, respectively. Plant treatments were conducted after one preconditioning month in a growth chamber as follows: (i) four foliar applications, 14, 7, and 2 days before inoculation and 10 days after inoculation; (ii) three irrigation applications, 7 and 2 days before inoculation and 10 days after inoculation. These schedules were established according to the manufacturer’s instructions. Exceptionally, hydrogen peroxide treatments were conducted three times per week from 1 week before inoculation to 2 weeks after inoculation for both foliar and irrigation applications according to the manufacturer’s recommendation. Nontreated and inoculated or noninoculated olive plants were also included as positive or negative controls, respectively. For each type of application, a randomized complete block design (three blocks) was used with 32 treatments and two controls (positive and negative) as independent variables and five replicated olive plants per treatment and block (34 × 3 × 5 = 510 plants per type of application; 1.020 plants in total).

#### Disease Severity Assessment

Disease severity (DS) was evaluated weekly for 12 weeks after inoculation using a 0 to 16 rating scale. This scale was designed to estimate the percentage of affected tissue by means four main categories: 0–25, 26–50, 51–75, and 76–100% of affected tissue, with four values per each category (0.25, 0.5, 0.75, and 1). Thus, scale values (X) represents the number of sixteenths of affected plant area (4 values per category × 4 categories), and they are linearly related to the percentage of affected tissue (Y) by the equation Y = 6.25X – 3.125 (Varo et al., 2018). DS data were used to calculate the relative area under the disease progress curve (RAUDPC) at the end of the experiment by the trapezoidal integration method (Campbell and Madden, 1990). In parallel, disease incidence (DI) and mortality were also assessed at the end of the experiment as the percentage of symptomatic or dead plants, respectively.

In addition, three symptomatic plants per treatment combination were randomly selected at the end of each experiment to confirm the infection of the pathogen by fungal isolation. Basal stems of the plants were washed under running tap water for 2 h. Subsequently, small fragments of the affected tissue were cut and surface sterilized by dipping them in a 10% solution of commercial bleach (Cl at 50 g L^–1^) for 1 min, air-dried on sterilized filter paper for 10 min, and plated onto APDA. Petri dishes were incubated as described before.

### Data Analyses

Data from this study were analyzed using Statistix 10.0 software (Analytical Software, 2013, Tallahassee, United States). All the experiments were conducted twice, and data from the two repetitions of each experiment were combined after checking for homogeneity of the experimental error variances by the *F* test (*P* ≥ 0.05). Subsequently, in any cases, data were tested for normality, homogeneity of variances, and residual patterns, and the square root transformation of the data was used when necessary. For the dual culture assays, ANOVA was conducted with “MGR” or “MGI” as the dependent variable, “BCAs” as the independent variable and each replicated Petri dish as a block. For the *in vitro* sensitivity tests, factorial ANOVA was conducted with “MGR” or “MGI” as dependent variables and “product,” “dose,” and their interaction as independent variables. Because the interaction “product” × “dose” was significant in both cases (*P* ≤ 0.0001), the differences in the effect on MGR and MGI among the evaluated products were analyzed separately for each dose as a completely randomized design. In any cases, treatments that showed 100% MGI were not included in the analysis. For the effect on MS viability, ANOVA was conducted with “MS density” or “MSI” as dependent variables and “soil treatment” as an independent variable. Soil treatments showing 100% MSI were not included in the analysis. For *in planta* experiments, ANOVA was conducted separately for each type of application (foliar or irrigation) with RAUDPC as the dependent variable and “treatment” as the independent variable. Treatments that did not show symptoms were not included in the analysis. Treatment means of the MGR or “MS concentration” were compared using Dunnett’s multiple comparison test with a control (MGR = 3.5 mm/day; MS concentration = 54.5 MS/g of soil) at *P* = 0.05. The treatment means of the MGI, MSI, RAUDPC and DS were compared according to Fisher’s protected LSD test at *P* = 0.05 (Steel and Torrie, 1985). Data on the final DI (% of affected plants) and mortality (% of dead plants) were analyzed by multiple comparisons for proportions tests at *P* = 0.05 (Zar, 2010). Additionally, the Pearson correlation coefficients (*r*) between the MGI and MSI of *V. dahliae*, and the RAUDPC of inoculated plants, were calculated using the average values of the three variables for each of the products evaluated at the irrigation dose (Table 1; *n* = 32).

## Results

### Effect on Mycelial Growth

#### Dual Culture Assay

Among the three BCAs tested in dual cultures, *B. amyloliquefaciens* PAB-24 and *Phoma* sp. ColPat-375 significantly reduced (*P* = 0.0071) the MGR of *V. dahliae* isolate V-180 in comparison with the control. Likewise, these two BCAs showed significantly (*P* = 0.0365) higher MGI values (47.3 ± 6.84 and 40.8 ± 15.2% for *B. amyloliquefaciens* PAB-24 and *Phoma* sp. ColPat-375, respectively) than that observed for *A. pullulans* AP08 (MGI = 19.5 ± 11.58%), which did not differ from the control (Table 2).

**TABLE 2:** Effect of three BCAs on mycelial growth of *Verticillium dahliae* in dual cultures.

Biological control agents	Isolate	MGR (mm day^–^^1^)^a,b^	MGI (%)^a,c^
*Aureobasidium pullulans*	AP08	2.8 ± 0.40	19.5 ± 11.58^b^
*Bacillus amyloliquefaciens*	PAB-24	1.8 ± 0.24*	47.3 ± 6.84^a^
*Phoma* sp.	ColPat-375	2.1 ± 0.53*	40.8 ± 15.2^a^
Control (*Verticillium dahliae*)	V180	3.5 ± 0.12	–

*Pictures below illustrate the mycelial growth of each BCA in dual cultures against *V. dahliae* isolate V180.*

*^*a*^Mycelial Growth Rate (MGR; mm day^–1^) and Mycelial Growth Inhibition (MGI; %) of *V. dahliae* were obtained after growing both the BCAs and *V. dahliae* in dual cultures on PDA at 24°C for 14 days in darkness. In any cases, data represents the average of eight replicated Petri dishes per BCA or control ± the standard error of the means.*

*^*b*^Means followed by an asterisk differ significantly from the “Control” according to Dunnett’s multiple comparison test at *P* = 0.05. ^*c*^Means followed by the same letter do not differ significantly according to Fisher’s protected LSD test at *P* = 0.05.*

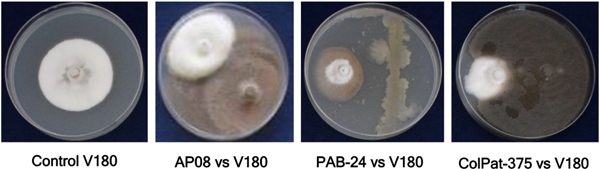

#### *In vitro* Sensitivity Test

The different products evaluated varied significantly (*P* ≤ 0.0001 in all cases) in their effect on the reduction of the MGR and MGI of *V. dahliae* depending on the dose tested. In general, *Bacillus subtilis*, copper chloride, copper sulfate, copper phosphites and fosetyl-Al inhibited the mycelial growth of *V. dahliae* by 100% at high and medium doses. In addition, *Bacillus subtilis* also inhibited the mycelial growth of *V. dahliae* by 100% at a low dose, whereas copper gluconate-1 only showed a 100% MGI at a high dose. On the other end, several products had no significant effect on the reduction of the MGR in comparison with the control at any dose tested. These products were seaweed extracts from *Laminaria digitata*, copper complex-1, organic carbon, hydrogen peroxide, and potassium silicate-1 and -2. In the remaining products, there was great variability in their effect on the MGR depending on the dose used. In general, the inhibition of mycelial growth by this group of products decreased when reducing the dose of the product, ranging from 77.7% for salicylic acid at the high dose to no significant effects of many products at the medium (*n* = 8) and low (*n* = 19) doses. Detailed MGR and MGI data for each product and dose combination are shown in Table 3.

**TABLE 3:** Effect of the commercial products evaluated in this study on mycelial growth of *Verticillium dahliae* isolate V180.

Products	Dose^a^					
	High		Medium		Low	
	MGR (mm day^–^^1^)^b,c^	MGI (%)^b,d^	MGR (mm day^–^^1^)^b,c^	MGI (%)^b,d^	MGR (mm day^–^^1^)^b,c^	MGI (%)^b,d^
Aluminum lignosulfonate	1.9 ± 0.40*	45.6 ± 11.56	2.3 ± 0.35*	34.9 ± 10.09	1.7 ± 0.09*	51.7 ± 2.57
Amino acids	1.6 ± 0.11*	55.2 ± 3.11	1.7 ± 0.07*	50.7 ± 1.93	2.3 ± 0.14*	33.3 ± 3.88
Amino acids + Cu	2.5 ± 0.04	27.9 ± 1.09	2.6 ± 0.03*	24.6 ± 0.79	2.8 ± 0.07	18.5 ± 2.11
Amino acids + N, P, K and S	1.6 ± 0.11*	54.3 ± 3.17	1.7 ± 0.27*	51.0 ± 7.87	2.4 ± 0.27*	32.3 ± 7.65
*Bacillus subtilis*	0.0 ± 0.00*	100 ± 0.00	0.0 ± 0.00*	100 ± 0.00	0.0 ± 0.00*	100 ± 0.00
Bioassimilable sulfur 1	1.2 ± 0.06*	66.2 ± 1.86	1.6 ± 0.05*	53.6 ± 1.45	2.7 ± 0.30	21.7 ± 8.71
Bioassimilable sulfur 2	1.6 ± 0.13*	53.5 ± 3.71	2.1 ± 0.06*	40.0 ± 1.80	2.2 ± 0.20*	37.0 ± 5.81
Chitosan	1.9 ± 0.26*	44.5 ± 7.49	2.0 ± 0.06*	43.2 ± 1.67	2.9 ± 0.27	17.5 ± 7.70
Copper chloride	0.0 ± 0.00*	100 ± 0.00	0.0 ± 0.00*	100 ± 0.00	2.9 ± 0.44	16.5 ± 12.61
Copper complexed 1	2.7 ± 0.08	23.8 ± 2.20	3.1 ± 0.32	13.8 ± 8.07	3.3 ± 0.17	5.5 ± 4.73
Copper complexed 2	2.1 ± 0.04*	39.8 ± 1.18	3.0 ± 0.09	13.09 ± 2.47	3.0 ± 0.17	15.0 ± 4.89
Copper gluconate 1	0.0 ± 0.00*	100 ± 0.00	1.8 ± 0.10*	47.8 ± 2.96	2.5 ± 0.13*	29.7 ± 3.77
Copper gluconate 2	1.9 ± 0.04*	46.7 ± 0.99	2.4 ± 0.22*	30.8 ± 6.23	2.9 ± 0.22	16.2 ± 5.63
Copper phosphite 1	0.0 ± 0.00*	100 ± 0.00	0.0 ± 0.00*	100 ± 0.00	2.1 ± 0.17*	41.0 ± 4.97
Copper phosphite 2	1.5 ± 0.10*	57.5 ± 2.68	2.2 ± 0.08*	38.0 ± 2.43	2.5 ± 0.10*	28.7 ± 2.87
Copper phosphite 3	1.2 ± 0.08*	65.4 ± 2.35	3.2 ± 0.07	8.6 ± 1.58	2.9 ± 0.18	18.5 ± 4.53
Copper sulfate	0.0 ± 0.00*	100 ± 0.00	0.0 ± 0.00*	100 ± 0.00	2.6 ± 0.27	25.0 ± 7.65
Fosetyl-Al	0.0 ± 0.00*	100 ± 0.00	0.0 ± 0.00*	100 ± 0.00	2.2 ± 0.10*	37.1 ± 2.78
Fungal extracts	1.1 ± 0.21*	69.8 ± 6.10	2.4 ± 0.13*	31.1 ± 3.69	2.7 ± 0.24	22.3 ± 6.79
Hydrogen peroxide	2.4 ± 0.32	31.2 ± 9.07	3.0 ± 0.25	16.3 ± 6.27	2.9 ± 0.23	16.5 ± 5.76
Organic carbón	2.7 ± 0.09	21.7 ± 2.59	3.1 ± 0.07	11.6 ± 1.94	3.1 ± 0.25	14.0 ± 5.72
Potassium phosphite 1	1.2 ± 0.06*	64.3 ± 1.76	2.5 ± 0.08*	27.0 ± 2.40	3.0 ± 0.06	15.0 ± 1.73
Potassium phosphite 2	1.8 ± 0.09*	48.4 ± 2.56	2.0 ± 0.08*	43.6 ± 2.27	2.7 ± 0.24	27.2 ± 1.48
Potassium phosphite 3	1.1 ± 0.11*	67.4 ± 3.19	2.2 ± 0.08*	37.1 ± 2.20	2.5 ± 0.05	23.7 ± 6.93
Potassium phosphite 4	1.4 ± 0.05*	58.7 ± 1.43	2.3 ± 0.11*	33.0 ± 3.32	2.9 ± 0.09	17.7 ± 2.42
Potassium silicate 1	3.3 ± 0.09	6.4 ± 2.53	3.2 ± 0.11	7.9 ± 3.27	3.2 ± 0.17	8.24 ± 4.34
Potassium silicate 2	3.3 ± 0.46	14.6 ± 8.52	2.9 ± 0.22	17.0 ± 5.84	2.4 ± 0.42*	31.6 ± 10.72
Salicylic acid	0.8 ± 0.27*	77.7 ± 7.64	2.0 ± 0.06*	44.0 ± 1.62	2.6 ± 0.24	24.9 ± 6.93
Seaweed extracts (*Laminaria digitata*)	3.4 ± 0.08	2.72 ± 2.02	3.8 ± 0.08	0.0 ± 0.00	3.5 ± 0.24	4.5 ± 4.04
**Control**	3.5 ± 0.12	−	3.5 ± 0.12	−	3.5 ± 0.12	−

*^*a*^Dose: high, the maximum dose recommended by the manufacturer of each product for its application by irrigation (Table 1); medium, 1/4 of the high dose; low, 1/16 of the high dose.*

*^*b*^Mycelial Growth Rate (MGR; mm day^–1^) and Mycelial Growth Inhibition (MGI; %) were obtained after growing *V. dahliae* isolate V180 onto amended or non-amended PDA at 24°C for 14 days in darkness. In both cases, data represents the average of eight replicated Petri dishes per product and dose combination or control ± the standard error of the means. Only for the MGR variable in the high dose experiment, the square root transformation of the data was performed to satisfy the ANOVA requirements for normality, homogeneity of variances and residual patterns.*

^*c*^For each dose, means of MGR followed by an asterisk (*) differ significantly from the “Control” according to Dunnett’s multiple comparison test at *P* = 0.05.

*^*d*^For each dose, significant differences between any treatment means of MGI are given by a critical value for means comparison [LSD_0.05_ = 12.5, 14.0, and 16.8 % for high, medium and low dose, respectively] according to Fisher’s protected LSD test at *P* = 0.05.*

#### Effect on Microsclerotia Viability

**FIGURE 1:**
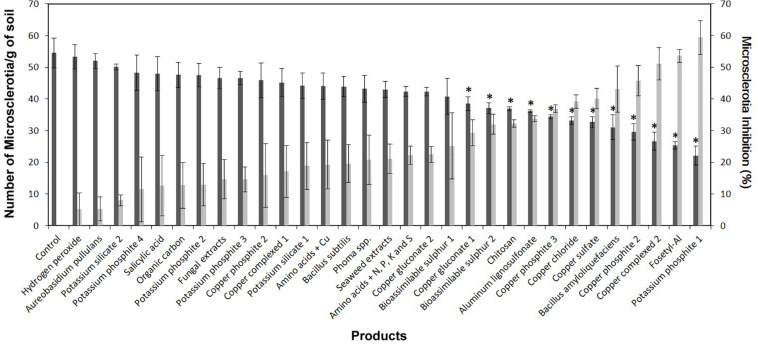
Effect of the evaluated products on the microsclerotia (MS) concentration [Number of MS/g of soil (dark gray columns)] and on the viability of MS [MS inhibition (MSI), % (light gray columns)] of *Verticillium dahliae* in naturally infested soil. Treatments were performed using the irrigation dose indicated for each product in Table 1. For each parameter, columns represent the average of six replicated plastic pots. Vertical bars represent the standard error of the means. For MS concentration, columns with an asterisk differ significantly from the control according to Dunnett’s multiple comparison test at *P* = 0.05. For MSI, significant differences between treatments were determined by a critical value for means comparison of 16.5% according to Fisher’s protected LSD test at *P* = 0.05.

The commercial products and BCAs evaluated showed significant differences among them for the MS viability of *V. dahliae* (*P* ≤ 0.0001 for both “MS concentration” and “MS inhibition” variables). The MS concentrations ranged from 22.1 ± 2.9 to 53.3 ± 3.8 MS g of soil^–1^ for potassium phosphite-1 and hydrogen peroxide, respectively. The following products showed significant differences in the reduction of MS concentration in comparison with the control (54.5 ± 4.7 MS g of soil^–1^): potassium phosphite-1, fosetyl-Al, copper complexed-2, copper phosphite-2, *B. amyloliquefaciens* (isolate PAB-024), copper sulfate, copper phosphite-3, copper chloride, aluminum lignosulfonate, chitosan, bioassimilable sulfur-2, and copper gluconate-1, with the “MS concentration” ranging between 38.5 ± 2.2 and 53.3 ± 3.8 MS g of soil^–1^. Likewise, the values of MS inhibition ranged from 59.4 ± 5.4 to 5.2 ± 5.2% for potassium phosphite-1 and hydrogen peroxide, respectively. The significant differences between products in terms of their effect on MSI were given by a critical value for means comparison of 16.5% according to Fisher’s protected LSD test at *P* = 0.05. It is interesting to note that our own BCAs evaluated in this study showed remarkable differences in their effect on MS viability. Only *B. amyloliquefaciens* isolate PAB-024 showed a significant reduction (43.1 ± 7.3%) in the MSI of *V. dahliae*, while the inhibition due to *Phoma* sp. isolate ColPat-375 (20.8 ± 7.7%) and *A. pullulans* isolate AP08 (5.3 ± 3.8%) did not differ significantly from that of the control (Figure 1). Linear correlation analysis showed that there was a low but significant correlation (*r* = 0.4603; *P* = 0.0080) between the MGI and MSI when products were evaluated at irrigation doses.

#### Effect on Verticillium Wilt Development in Olive Plants

The factorial ANOVA for the 32 evaluated products with the two types of applications (foliar and irrigation) showed a non-significant effect of application type (*P* = 0.4533 and *P* = 0.9138 for RAUDPC and DS, respectively), but the variable “product” and the interaction between product and type of application were significant (*P* ≤ 0.0001 in any cases for both dependent variables). Thus, *in planta* experiments were analyzed separately for each type of application, foliar and irrigation, and the data are shown in Tables 4, 5, respectively.

**TABLE 4:** Disease-related parameters for olive plants grown in artificially infested substrate with the defoliating *Verticillium dahliae* isolate V180 and treated with the evaluated products by foliar application^*a*^.

Products	Incidence (%)^b^	Mortality (%)^b^	Disease severity (%)^c^	RAUDPC (%)^d^
Negative control	0.0	0.0	0.0 ± 0.00	0.0 ± 0.00
Positive control	100.0	100.0	100.0 ± 0.00	100.0 ± 0.00
Aluminum lignosulfonate	93.3	0.0	60.8 ± 11.49	66.5 ± 13.55
Amino acids	64.3	9.5	29.0 ± 4.92	43.3 ± 13.81
Amino acids + Cu	78.6	19.1	50.6 ± 16.18	66.5 ± 24.52
Amino acids + N, P, K and S	69.2	0.0	34.7 ± 12.65	38.0 ± 18.86
*Aureobasidium pulullans*	80.4	14.0	42.7 ± 2.91	36.4 ± 2.72
*Bacillus amyloliquefaciens*	100.0	44.4	83.5 ± 6.89	65.9 ± 6.39
*Bacillus subtilis*	75.0	22.3	68.9 ± 26.31	108.2 ± 47.01
Bioassimilable sulfur 1	77.3	42.9	44.5 ± 10.46	79.0 ± 19.61
Bioassimilable sulfur 2	53.8	20.5	29.0 ± 4.51	49.7 ± 17.84
Chitosan	100.0	53.3	86.4 ± 4.55	102.1 ± 7.11
Copper chloride	93.3	0.0	42.6 ± 9.49	50.9 ± 5.40
Copper complexed 1	100.0	35.6	76.1 ± 8.93	60.9 ± 11.91
Copper complexed 2	87.9	35.6	91.8 ± 12.39	72.2 ± 13.90
Copper gluconate 1	86.7	8.9	32.4 ± 5.11	48.5 ± 6.70
Copper gluconate 2	91.7	15.6	61.4 ± 6.28	66.5 ± 13.55
Copper phosphite 1	100.0	80.0	104.6 ± 15.03	99.4 ± 18.05
Copper phosphite 2	64.3	38.1	52.5 ± 8.71	80.8 ± 9.17
Copper phosphite 3	87.9	41.1	81.3 ± 9.81	64.6 ± 8.48
Copper sulfate	86.7	19.1	65.8 ± 6.39	67.6 ± 12.07
Fosetyl-Al	93.3	8.9	32.4 ± 16.20	42.8 ± 17.88
Fungal extracts	91.7	44.4	51.4 ± 12.87	60.3 ± 19.60
Hydrogen peroxide	100.0	0.0	42.1 ± 4.65	47.1 ± 4.67
Organic carbon	86.0	13.7	48.0 ± 12.31	46.7 ± 16.3
*Phoma* spp.	75.0	0.0	34.1 ± 13.31	35.6 ± 17.56
Potassium phosphite 1	75.0	22.3	55.4 ± 20.33	62.3 ± 28.66
Potassium phosphite 2	78.6	9.5	39.2 ± 9.69	48.5 ± 15.25
Potassium phosphite 3	71.7	18.4	46.0 ± 14.54	65.1 ± 16.25
Potassium phosphite 4	78.6	35.6	99.2 ± 11.80	81.5 ± 14.88
Potassium silicate 1	93.3	17.8	48.3 ± 9.14	52.8 ± 11.24
Potassium silicate 2	83.3	22.3	70.3 ± 11.73	124.6 ± 20.98
Salicylic acid	72.4	14.0	36.4 ± 8.44	46.3 ± 11.11
Seaweed extracts (*L. digitata*)	78.6	9.5	45.5 ± 2.56	49.3 ± 4.26
LSD_0.__05_	19.7^e^	21.6^e^	30.4^f^	45.1^f^

*^*a*^Olive plants were treated several times before and after inoculation and disease parameters were assessed weekly for 12 weeks after inoculation with *V. dahliae*.*

*^*b*^Percentage of symptomatic plants (Incidence) or dead plants (Mortality) 12 weeks after planting in the infested substrate with *V. dahliae* isolate V180 (*n* = 30).*

*^*c*^Final disease severity ± Standard Error of the means (SE) 12 weeks after planting in the infested substrate with *V. dahliae* isolate V180. Disease severity was assessed using a rating scale of 0 to 16 (0 = no lesions, 16 = 94–100% of affected plant tissues).*

*^*d*^Relative area under the disease progress curve (RAUDPC) ± SE developed over the assessment period.*

*^*e*^Critical value for means comparison according to Zar’s multiple comparisons for proportions test at *P* = 0.05 (Zar, 2010).*

*^*f*^Critical value for means comparison according to Fisher’s protected LSD test at *P* = 0.05 (Steel and Torrie, 1985).*

**TABLE 5:** Disease-related parameters for olive plants grown in artificially infested substrate with the defoliating *Verticillium dahliae* isolate V180 and treated with the evaluated products by irrigation^a^.

Products	Incidence (%)^b^	Mortality (%)^b^	Disease severity (%)^c^	RAUDPC (%)^d^
Negative control	0.0	0.0	0.0 ± 0.00	0.0 ± 0.00
Positive control	100.0	100.0	100.0 ± 0.00	100.0 ± 0.00
Aluminum lignosulfonate	80.0	0.0	36.9 ± 7.26	28.7 ± 3.09
Amino acids	71.4	19.1	49.4 ± 9.39	54.8 ± 22.12
Amino acids + Cu	91.7	0.0	46.9 ± 12.84	53.4 ± 15.58
Amino acids + N, P, K and S	92.3	0.0	51.9 ± 7.52	55.7 ± 14.35
*Aureobasidium pulullans*	77.3	4.6	32.1 ± 1.64	25.6 ± 2.62
*Bacillus amyloliquefaciens*	80.0	17.8	41.5 ± 3.28	27.3 ± 6.55
*Bacillus subtilis*	83.3	44.4	64.6 ± 17.67	89.4 ± 14.35
Bioassimilable sulfur 1	100.0	66.7	69.7 ± 14.35	96.3 ± 22.72
Bioassimilable sulfur 2	78.6	0.0	45.5 ± 8.93	46.5 ± 6.47
Chitosan	93.3	17.8	76.7 ± 12.80	77. 8 ± 16.96
Copper chloride	100.0	100.0	136.4 ± 0.01	196.1 ± 0.45
Copper complexed 1	100.0	35.6	71.6 ± 30.13	50.8 ± 23.60
Copper complexed 2	91.7	77.8	65.8 ± 9.78	100.0 ± 12.95
Copper gluconate 1	86.7	8.9	42.0 ± 8.48	40.1 ± 15.08
Copper gluconate 2	85.9	4.5	41.5 ± 3.28	35.8 ± 6.41
Copper phosphite 1	100.0	53.3	102.9 ± 8.60	155.1 ± 12.59
Copper phosphite 2	78.6	19.1	43.8 ± 10.43	71.7 ± 38.3
Copper phosphite 3	51.5	2.8	22.7 ± 2.87	20.6 ± 5.17
Copper sulfate	100.0	44.4	77.8 ± 11.91	67.8 ± 7.11
Fosetyl-Al	100.0	93.3	88.6 ± 4.92	176.0 ± 14.26
Fungal extracts	93.3	80.0	70.7 ± 12.85	107.2 ± 44.35
Hydrogen peroxide	100.0	0.0	58.0 ± 9.49	53.3 ± 13.11
Organic carbon	62.6	14.7	37.8 ± 7.65	42.1 ± 8.48
*Phoma* spp.	100.0	33.3	71.0 ± 27.41	71.9 ± 33.87
Potassium phosphite 1	66.7	11.1	43.3 ± 17.93	48.5 ± 22.33
Potassium phosphite 2	42.9	19.1	21.0 ± 16.77	46.5 ± 39.13
Potassium phosphite 3	70.9	10.5	37.8 ± 7.17	22.3 ± 11.58
Potassium phosphite 4	100.0	38.1	76.1 ± 11.34	79.9 ± 22.68
Potassium silicate 1	93.3	0.0	40.9 ± 7.43	37.1 ± 6.55f
Potassium silicate 2	50.0	0.0	26.3 ± 3.76	60.4 ± 18.11
Salicylic acid	85.1	17.6	55.7 ± 6.42	67.0 ± 9.63
Seaweed extracts (*L. digitata*)	78.6	19.1	29.8 ± 9.29	45.5 ± 14.26
LSD_0.05_	19.5^e^	22.7^e^	33.2^f^	54.0^f^

*^*a*^Olive plants were treated several times before and after inoculation and disease parameters were assessed weekly for 12 weeks after inoculation with *V. dahliae*.*

*^*b*^Percentage of symptomatic plants (Incidence) or dead plants (Mortality) 12 weeks after planting in the infested substrate with *V. dahliae* isolate V180 (*n* = 30).*

*^*c*^Final disease severity ± Standard Error of the means (SE) 12 weeks after planting in the infested substrate with *V. dahliae* isolate V180. Disease severity was assessed using a rating scale of 0 to 16 (0 = no lesions, 16 = 94–100% of affected plant tissues).*

*^*d*^Relative area under the disease progress curve (RAUDPC) ± SE developed over the assessment period.*

*^*e*^Critical value for means comparison according to Zar’s multiple comparisons for proportions test at *P* = 0.05 (Zar, 2010).*

*^*f*^Critical value for means comparison according to Fisher’s protected LSD test at *P* = 0.05 (Steel and Torrie, 1985).*

Nontreated control plants inoculated with *V. dahliae* isolate V180 showed typical symptoms of VWO approximately 30 days after inoculation, with a progressively increasing DS until reaching a final DI of 100% at 3 months after inoculation. Although the factorial ANOVA did not show a significant effect of the type of application, the irrigation treatments seemed more effective in reducing disease progress (RAUDPC) and DS than foliar treatments. Regarding foliar treatments, a significant effect was observed between products (*P* = 0.0014) on the RAUDPC, with 14 out of the 32 products evaluated showing a significant effect in the reduction of disease progress in comparison with the control. The RAUDPC values of these products ranged from 52.8 ± 11.2 to 35.9 ± 17.6% for potassium silicate-1 and *Phoma* sp. (isolate ColPat-375), respectively. In addition, the BCA *A. pullulans* (isolate AP08) was the second most effective product (RAUDPC = 36.4 ± 2.7) after *Phoma* sp. Potassium silicate-2 showed a remarkably higher RAUDPC (124.6 ± 21.0%) than the control (RAUPDC = 100%), probably due to its phytotoxic effect *in planta* with spray application. Considering the final DS, copper phosphite-1 (104.6 ± 15.0%) and potassium phosphite-4 (99.2 ± 11.8%) were the least effective products, while amino acids and bioassimilable sulfur-2 were the most effective products, with a final DS of 29.0 ± 14.5 and 29.0 ± 4.5%, respectively. Values of DI among the evaluated products ranged from 100% for *B. amyloliquefaciens*, chitosan, copper complex-1, copper phosphite-1 and hydrogen peroxide to 53.8% for bioassimilable sulfur-2. Finally, values of plant mortality among the evaluated products ranged from 80.0% for copper phosphite-1 to 0.0% for aluminum lignosulfonate, amino acids+N,P,K,S, copper chloride, hydrogen peroxide and *Phoma* sp. (Table 4).

On the other hand, a significant effect was also observed on the RAUDPC between products (*P* ≤ 0.0001) when they were applied by irrigation, with 10 out of the 32 products evaluated showing a significant effect in the reduction of disease progress in comparison with the control. Among the most effective products, we found RAUDPC values ranging from 45.5 ± 14.3 to 20.6 ± 5.2% for seaweed extracts (*L. digitata*) and copper phosphite-3, respectively. However, copper chloride, fosetyl-Al and copper phosphite-1 showed a significantly higher RAUDPC in comparison with the control, ranging between 196.1 ± 0.5, 176.0 ± 14.3 and 155.1 ± 12.6, respectively. These higher RAUDPC values could also be attributed to their phytotoxic effects in potted plants. Based on the final DS, copper chloride (136.4 ± 0.01%), fosetyl-Al (88.6 ± 4.9%) and copper phosphite-1 (102.9 ± 8.65%) were also the least effective products, whereas copper phophite-3 (22.7 ± 2.9%) and potassium phosphite-2 (21.02 ± 16.8%) were the most effective products. Nine out of the 32 evaluated products resulted in 100% DI, but copper phosphite-3, potassium phosphite-2 and potassium silicate-2 resulted in DI values of approximately 50%. Finally, seven out of the 32 products evaluated resulted in null mortality, whereas fosetyl-Al (93.3%) and copper chloride (100%) resulted in the highest values (Table 5). Linear correlation analysis showed that there was not significant correlation between the MGI and RAUDPC (*r* = 0.2424; *P* = 0.2139), and between MSI and RAUDPC (*r* = 0.0608; *P* = 0.7586) when products were evaluated at irrigation doses.

The pathogen was successfully reisolated from the basal stem tissues of all selected symptomatic plants (consistency of isolation = 60-90%), confirming the infection by *V*. *dahliae* in the inoculated plants.

## Discussion

Plant biostimulants are characterized by a wide and non-precise definition, but they never can be defined as fertilizers since they do not provide nutrients directly to the plant. Likewise, a broad range of substances or mixtures of substances, including enzymes, proteins, amino acids, nutrients, phenols, humic and fulvic acid, protein hydrolases, and microorganisms (fungi and bacteria), are included under the term biostimulants. Despite the diversity of products grouped as biostimulants, they must all improve the condition of treated plants but not cause adverse side effects in any case (Drobek et al., 2019). Therefore, considering all these aspects and according to the recent European Regulation (EU) 2019/1009 on biostimulants, most of the products (22 out of 32) evaluated in this study were selected to determine their effect against VWO. In addition, chemicals such as fosetyl-Al were also included in this study since they have been grouped as HPDI by Fungicide Resistance Action Committee (FRAC), as well as chitosan and salicylic acid. Altogether, this study represents novel and relevant information on the biological control of VWO using BCAs, plant biostimulants and HPDIs.

Concerning the BCAs from our own collection (non-commercial products), the bacterium *B. amyloliquefaciens* isolate PAB-024 and the fungus *Phoma* sp. isolate ColPat-375 significantly reduced the MGR, whereas only *B. amyloliquefaciens* reduced significantly the MS concentration of *V. dahliae* in comparison with the controls. However, the fungus *A. pullulans* (isolate AP08) showed no effect on the pathogen. Recently, Varo et al. (2016) evaluated a wide diversity of microorganisms against *V. dahliae*, showing similar results for the MGI of the pathogen for another *Phoma* sp. isolate. However, our results on the effect of *B. amyloliquefaciens* are in contrast with those obtained by these same authors since our isolate of *B. amyloliquefaciens* showed a remarkably higher MGI of *V. dahliae* in comparison with that obtained by Varo et al. (2016). It is interesting to note that the isolate of *B. amyloliquefaciens* used in this study also showed a high effect on disease reduction *in planta* when it was applied by irrigation. Thus, *B. amyloliquefaciens* isolate PAB-024 was highly effective in reducing pathogen development (MGR and MSI) as well as in reducing the disease *in planta*. These results suggest that this BCA could act as a direct antagonist of the pathogen. In contrast, *A. pullulans* isolate AP08 was highly effective in the inhibition of the disease in olive plants with both foliar and irrigation applications, but it showed no effect on either the MGI or MSI of *V. dahliae*. The differences obtained for the effect of *A. pullulans* between *in vitro* and *in planta* experiments conducted in the present study suggest that this BCA could act as a HPDI instead of as an antagonist of *V. dahliae*. In addition, the significant effect on the reduction of disease severity observed with foliar applications of *Phoma* sp. agrees again with that obtained by Varo et al. (2016), who also showed an important effect on the reduction of disease severity *in planta* using another *Phoma* sp. isolate.

The effect of the remaining 29 selected products (commercial products) on mycelial growth and the MSI of *V. dahliae* was evaluated by *in vitro* sensitivity tests to check whether the commercial products had any fungicidal effect interfering with the growth of the pathogen. Seventy-six percent of the commercial products significantly reduced the MGR of *V. dahliae* in comparison with the control. However, only 12 out of the 32 products evaluated were able to significantly reduce the MS concentration of the pathogen in naturally infested soil. In this case, the lack of direct toxicity of some products is not a reason *a priori* to discard them as potential effective products against the disease since they could act as HPDIs (Llorens et al., 2017b). Likewise, all the products were evaluated *in planta* not only to determine their effect on disease development but also to evaluate their possible phytotoxic effects. It is worth mentioning that no correlation was observed in the effectiveness of the products between *in vitro* and *in planta* experiments. This lack of correlation could be due to their effect as HPDIs.

In general, irrigation treatments were more effective than foliar treatments. This result is in concordance with that obtained in previous studies evaluating the effect of plant biostimulants on different pathosystems, where irrigation treatments were always more effective in enhancing plant innate defenses than foliar treatments (Llorens et al., 2017a, 2019; González-Hernández et al., 2018).

Copper-based products, including copper chloride, copper sulfate, complexed copper and copper gluconates, were among the most effective products in the reduction of MGR and MS concentrations of *V. dahliae*. However, they did not show an important effect on the reduction of disease severity *in planta* with any type of application (foliar or irrigation). Only copper chloride showed high levels of phytotoxicity among the copper-based products tested. These differences in their effect between *in vitro* and *in planta* experiments could be attributed to the protective mode of action of copper (Roca et al., 2007). Copper-based products do not penetrate plant tissues but prevent pathogen growth by forming a protective surface sheet, thus preventing the infection from spreading to healthy tissues. For this reason, although copper-based products have been traditionally used to prevent the main olive foliar diseases due to their high effectiveness (Roca et al., 2007), their use as fungicides against VWO is limited because they are not able to penetrate into the vascular tissues of the affected plants. However, the current formulations of complexed copper has been mentioned as a potential alternative to prevent plant infections in other pathosystems since this kind of formulation could enhance plant innate defenses even before the pathogen infection event (González-Hernández et al., 2018). Phytotoxicity was shown in many plants treated with copper chloride, probably because the product tested was not a commercially formulated fungicide. Thus, its higher solubility in comparison with commercial phytosanitary formulations may have led to the development of severe phytotoxicity.

On the other hand, phosphite salts are emerging as novel and relevant plant biostimulants. They have not been proven to have a direct effect on plant nutrition, but they are able to markedly improve crop yield and quality and stimulate both biotic and abiotic stress responses in crops (Gómez-Merino and Trejo-Téllez, 2015). In fact, two out of the six phosphites evaluated in this study (copper phosphite-3 and potassium phosphite-1) were the most effective products against VWO *in planta* when they were applied by irrigation. Their high effectiveness in reducing the DS and mortality of olive plants infected by *V. dahliae* make them promising candidates preventing VWO. It is worth mentioning that important differences between products belonging to the same chemical group (i.e., complexed copper, copper phosphites, potassium phosphites, or silicates, etc.) were observed on the effect of fungal or disease development in *in vitro* or *in planta* experiments, respectively. In this sense, it is remarkable that irrigation treatments with copper phosphite-3 showed the highest levels of disease reduction *in planta*, whereas copper phosphite-1 resulted in high levels of phytotoxicity. This aspect could be attributed to the different formulations of the products made by the respective manufacturers, and it should always be considered in the selection of the appropriate products.

A wide diversity in the response to the pathogen and disease reduction was observed for the rest of the commercial products tested. Some products, such as fosetyl-Al or fungal extracts or potassium silicate-2, showed phytotoxicity when they were applied by irrigation or foliar treatments, respectively. In addition to the BCAs tested in this study, the only commercial BCA evaluated (commercial strain of *B. subtilis*) was the most effective biological treatment against the pathogen *in vitro* but had a markedly minor effect on reducing DS *in planta*. The results suggest that this BCA acts directly on the pathogen, although we cannot discard its potential effect as a HPDI in other pathosystems. Salicylic acid showed an intermediate disease reduction *in planta* when it was sprayed, but no significant difference was observed in comparison with the control when it was applied by irrigation. Similar results regarding foliar treatments were obtained by Gharbi et al. (2016), who showed that foliar applications with salicylic acid before inoculation were able to significantly reduce the progression of VWO in potted olive plants. Finally, seaweed extract showed intermediate effectiveness in disease reduction for both foliar and irrigation applications *in planta*, but it had a null effect on the MGI and MSI of *V. dahliae*. Although it was not among the most effective products of this study against VWO, the null toxicity of seaweed to the fungi makes it a potential plant biostimulant against wilt diseases. In fact, the use of seaweeds in the biological control of VWO has been previously investigated because they can act as elicitors of phenylalanine ammonia-lyase (PAL) and lignin in olive (Montes-Osuna and Mercado-Blanco, 2020). In this way, Salah et al. (2018) demonstrated that applications of seaweed extracts including alginate, carrageenan, laminarin or ulvan in olive twigs increased the PAL activity, which was correlated with the lignin content in the treated twigs. In addition, treated twigs showed a significant reduction in vascular discoloration caused by *V. dahliae* (Salah et al., 2018).

In summary, our results suggest that microorganisms from our own collection are among the most effective treatments for the reduction of VWO *in planta*. *Phoma* sp. isolate ColPat-375 and *A. pullulans* isolate AP08 were most effective when applied with foliar application. On the other hand, *B. amyloliquefaciens* isolate PAB-024 and *A. pullulans* isolate AP08 were among the most effective irrigation treatments after potassium phosphite-3 and copper phosphite-3. As we mentioned throughout the discussion, these BCAs or plant biostimulants could present different modes of action, such as antagonism or host resistance induction. However, the methodology used in this study was not sufficient to determine the modes of action of the different products. Therefore, further research is needed to select the most effective products and determine their mode of action by means of biochemical tools. In this way, monitoring the main parameters involved in plant resistance, such as quantification of H_2_O_2_ and callose deposition, and evaluation of hormones related to plant defense in olive tissues after treatments will be necessary to elucidate their role as HPDIs. Therefore, the present work will be useful to select better candidates for future studies on biocontrol, contributing significantly to new insights into the current challenge of the biological control of VWO.

## Data Availability Statement

The raw data supporting the conclusions of this article will be made available by the authors, without undue reservation.

## Author Contributions

AT and CA-B conceived and designed the study. AL-M and CA-B performed the laboratory work. AL-M analyzed the data and wrote and edited the manuscript. CA-B and AT reviewed the manuscript. AT acquired the funding support. All authors contributed to the article and approved the submitted version.

## Conflict of Interest

The authors declare that the research was conducted in the absence of any commercial or financial relationships that could be construed as a potential conflict of interest.
